# A Promising Preoperative Prediction Model for Microvascular Invasion in Hepatocellular Carcinoma Based on an Extreme Gradient Boosting Algorithm

**DOI:** 10.3389/fonc.2022.852736

**Published:** 2022-03-04

**Authors:** Weiwei Liu, Lifan Zhang, Zhaodan Xin, Haili Zhang, Liting You, Ling Bai, Juan Zhou, Binwu Ying

**Affiliations:** ^1^Department of Laboratory Medicine, West China Hospital, Sichuan University, Chengdu, China; ^2^Department of Gastroenterology and Hepatology, West China Hospital, Sichuan University, Chengdu, China; ^3^Department of Liver Surgery & Liver Transplantation Center, West China Hospital, Sichuan University, Chengdu, China

**Keywords:** microvascular invasion, non-invasive predictive models, machine learning, extreme gradient boosting (XGBoost), hepatocellular carcinoma

## Abstract

**Background:**

The non-invasive preoperative diagnosis of microvascular invasion (MVI) in hepatocellular carcinoma (HCC) is vital for precise surgical decision-making and patient prognosis. Herein, we aimed to develop an MVI prediction model with valid performance and clinical interpretability.

**Methods:**

A total of 2160 patients with HCC without macroscopic invasion who underwent hepatectomy for the first time in West China Hospital from January 2015 to June 2019 were retrospectively included, and randomly divided into training and a validation cohort at a ratio of 8:2. Preoperative demographic features, imaging characteristics, and laboratory indexes of the patients were collected. Five machine learning algorithms were used: logistic regression, random forest, support vector machine, extreme gradient boosting (XGBoost), and multilayer perception. Performance was evaluated using the area under the receiver operating characteristic curve (AUC). We also determined the Shapley Additive exPlanation value to explain the influence of each feature on the MVI prediction model.

**Results:**

The top six important preoperative factors associated with MVI were the maximum image diameter, protein induced by vitamin K absence or antagonist-II, α-fetoprotein level, satellite nodules, alanine aminotransferase (AST)/aspartate aminotransferase (ALT) ratio, and AST level, according to the XGBoost model. The XGBoost model for preoperative prediction of MVI exhibited a better AUC (0.8, 95% confidence interval: 0.74–0.83) than the other prediction models. Furthermore, to facilitate use of the model in clinical settings, we developed a user-friendly online calculator for MVI risk prediction based on the XGBoost model.

**Conclusions:**

The XGBoost model achieved outstanding performance for non-invasive preoperative prediction of MVI based on big data. Moreover, the MVI risk calculator would assist clinicians in conveniently determining the optimal therapeutic remedy and ameliorating the prognosis of patients with HCC.

## 1 Introduction

Hepatocellular carcinoma (HCC) is one of the most common malignancies and the third leading cause of cancer-related death worldwide (1). Surgical resection is one of the predominant treatments for early-stage HCC; however, the high incidence of postoperative recurrence and metastasis largely threatens the long-term survival of patients (2). Microvascular invasion (MVI), the embolus of cancer cells with micro-metastasis in liver vessels, is an independent prognostic factor for recurrence and metastasis in HCC (3). Recently, an increasing number of studies (4, 5) have shown that a precise surgical approach and timely postoperative adjuvant therapy for patients with HCC and MVI could reduce recurrence and improve survival.

Patients with HCC and MVI have been demonstrated to achieve better prognosis through anatomical resection than through non-anatomical resection (6). In addition, scholars have suggested that patients with HCC and MVI should be treated with wide margin resection rather than narrow margin resection, as it achieves better relapse-free survival (7). Besides, surgical resection provides better tumor control than radiofrequency ablation (RFA) treatment in patients with small HCC, especially those with a high risk of MVI (8). MVI status is crucially important for clinicians to choose the optimal therapy, while MVI can only be confirmed by postoperative histopathological examination; therefore, preoperative prediction of MVI is urgent.

Early studies have focused on blood biomarkers that can predict MVI; among these, α-fetoprotein (AFP) is considered one of the most notable biomarkers (9). However, its predictive efficacy for MVI was poor in univariate analysis (10, 11), while the combination of multiple biomarkers showed greater potential (12, 13). With the development of big-data-driven approaches, machine learning (ML) has been extensively used in various diseases, such as cardiac abnormalities (14), pulmonary diseases (15), neurological disorders (16), and oncology (17–20), showing great ability in prediction model construction. ML algorithms demonstrate the advantages of robust feature selection and the ability to identify clinically important risks among patients, are dedicated to finding complex patterns in big data with high accuracy and are suitable for constructing predictive models from numerous multidimensional factors, especially non-linear complex data.

However, different ML algorithms have their own advantages and disadvantages. Recently, Deng et al. combined the neutrophil-to-lymphocyte ratio, tumor size, and AFP to establish a nomogram for predicting MVI in 513 patients with HCC, but the sensitivity and specificity of the model were only 61.64% and 71.53%, respectively (21). Lei et al. constructed a nomogram to predict MVI in 1004 patients with HCC, but the high false-positive (23.4%) and false-negative rates (26.5%) still need to be considered when it is applied in clinical decision-making (22). These models were based on logistic regression algorithms; although simple to construct, they were prone to underfitting, and the clinical application accuracy was not ideal. Therefore, more ML algorithms have been applied to predict the occurrence of MVI and achieve better performance. Chen et al. proposed an MVI prediction model by integrating blood tests based on a deep learning method with concordance indexes of 0.9341 and 0.9052 in the training and validation cohorts, respectively (23). Additionally, the inclusion of radiomic features and multi-omics data improved the model’s predictive performance of MVI. Xu et al. integrated radiomics, clinical features, and liver and renal function indicators and developed a multivariate logistic regression MVI prediction model in 495 patients with HCC, with an area under the receiver operating characteristic curve (AUC) of 0.889 (24). However, these radiomic features require sophisticated techniques and experts and are not easy to popularize. Overall, the sample size of previous studies was small, ranging from 150 to 1004 patients (24, 25), and it was demonstrated that the robustness of the model based on big data was better than that based on small data (26). Therefore, it is necessary to establish an MVI prediction model with reliable and excellent performance using conveniently available clinical indicators and big data.

The noninvasive preoperative diagnosis of MVI in HCC is vital for precise surgical decision-making and patient prognosis. In this study, we attempted to use multiple ML algorithms to develop a preoperative MVI prediction model and select an optimal one, based on the big data of patients with HCC at the West China Hospital, from multidimensional and conveniently available variables. Simultaneously, we quantified and explained the important variables related to MVI, visually exhibiting them using the Shapley Additive exPlanation (SHAP) algorithm. Remarkably, to make it more convenient in clinical situations, we created an MVI risk online calculator for clinicians to assist in precise HCC treatment visually and operationally.

## 2 Methods

### 2.1 Ethics and Statements

This study was approved by the Institutional Ethics Committee of the West China Hospital, Sichuan University [number: 2019 (203)].

### 2.2 Study Design and Patients

**Figure 1:**
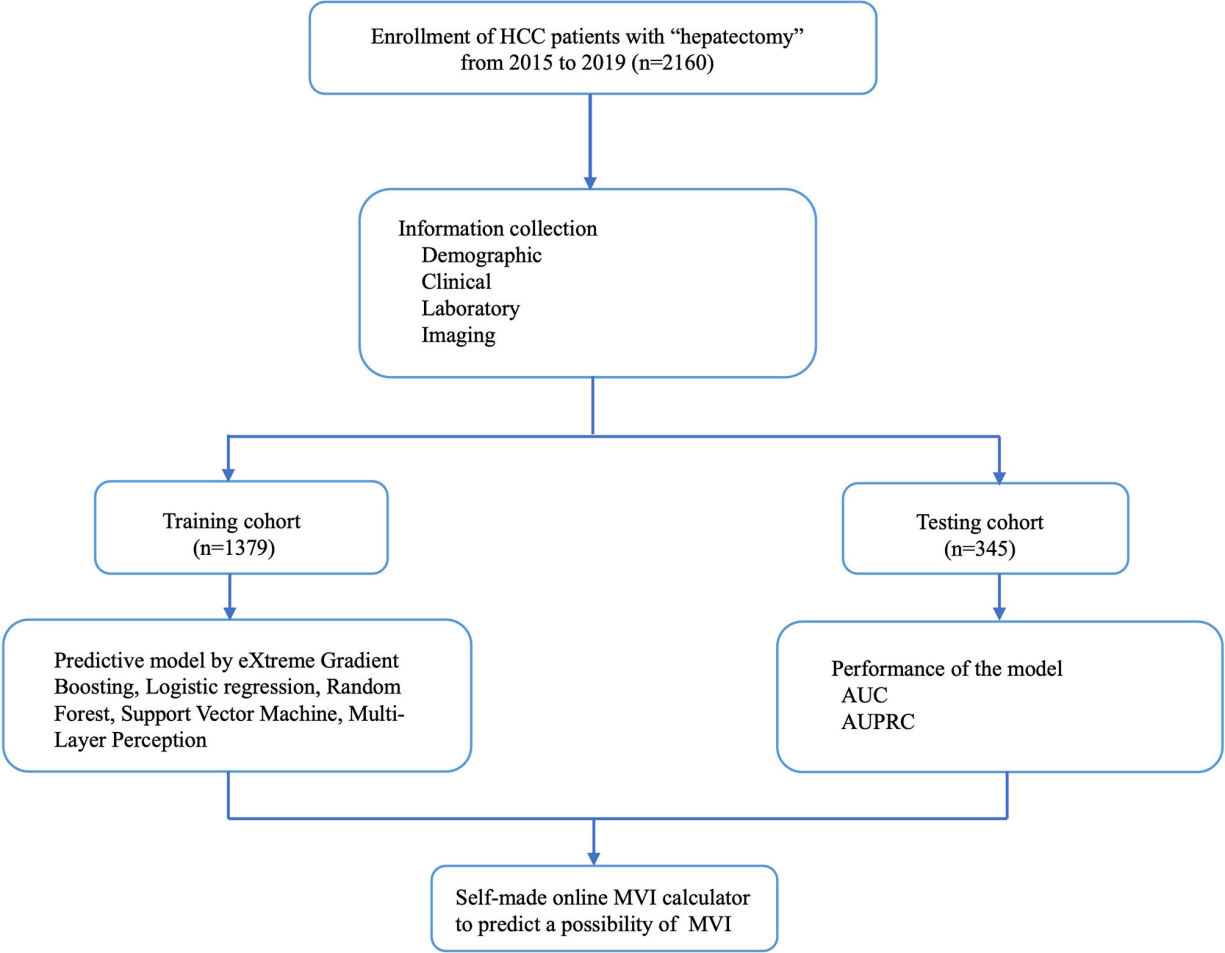
Flow chart of patient selection.

Patients with HCC who underwent surgery at the West China Hospital between January 2015 and June 2019 were retrospectively enrolled (**Figure 1**). The inclusion criteria were as follows: 1) patients who had undergone hepatectomy or liver transplantation for the first time and were pathologically diagnosed with HCC alone, regardless of whether they had received transcatheter arterial chemoembolization (TACE) before; and 2) the Guidelines for Diagnosis and Treatment of Primary Liver Cancer in China (2017 Edition) (27) were used as diagnostic criteria. The exclusion criteria were as follows: 1) Patients with HCC with recurrence who had previously undergone hepatectomy or RFA; 2) HCC with macroscopic invasion; 3) Patients with HCC and tumors at other sites; and 4) Patients with HCC and other tumors, such as bile duct adenocarcinoma, intrahepatic cholangiocarcinoma, and sarcoma.

MVI diagnosis has relied on the judgment of two or more experienced pathologists based on seven-point sampling (28) to ensure MVI detection ability since 2015 in our hospital; hence, we used data from 2015.

### 2.3 Preoperative Examination and Clinicopathologic Variables

In total, 88 indicators were extracted from Electronic Health Records (EHR) of all patients. All of the indicators were all collected within 1 month before surgery, including three categories of characteristics: 1) demographic data: age, gender, height, weight, body mass index, ethnicity, preoperative TACE (yes or no), hepatitis B, and hepatitis C; 2) imaging features: single or multiple tumors, maximum diameter of the tumors, imaging cirrhosis, tumor margin, etc.; 3) laboratory examination results: routine blood test results, biochemical test results (hepatic function indexes, kidney function indexes, glucose, etc.), routine coagulation test results (activated partial thromboplastin time [APTT], prothrombin time, etc.), hepatitis B virus (HBV)-DNA load and tumor markers (AFP, carbohydrate antigen, carcinoembryonic antigen, cancer antigen 125, protein induced by vitamin K absence or antagonist-II [PIVKA-II]). The details of all indicators are listed in **Supplemental Table 1**.

### 2.4 Statistical Analysis

Continuous variables with normal distribution are expressed as mean ± standard deviation and were compared between the MVI and non-microvascular invasion (NMVI) groups using Student’s t-test. Non-normal variables were analyzed using the Kruskal-Wallis rank sum test. Categorical variables are expressed as frequency (%), and chi-square tests or Fisher exact tests were applied to these data, as appropriate. All statistical analyses were performed using Python (version 3.7.9), and *p* < 0.05 indicated statistical significance.

### 2.5 Machine Learning Models Establishment

The patients with HCC in this study were randomly divided into a training cohort and a validation cohort at a ratio of 8:2. To overcome the imbalance in the data, an under-sampling approach was applied (29). We attempted to develop MVI predictive models using five ML algorithms: logistic regression (LR), random forest (RF), support vector machine (SVM), multilayer perception (MLP), and extreme gradient boosting (XGBoost). LR involves modeling the relationship between explanatory variables and the log odds of a binary outcome by employing the maximum likelihood algorithm (30). RF, a tree-like model, integrates multiple decision trees through major voting, reducing variance, and increasing robustness (31). SVM is a computational algorithm that separates binary labeled data based on a line to realize the maximum distance between the labeled data using hinge loss to calculate the empirical risk (32). An MLP is typically built into structured node groups with activation functions and connection weights that mimic the behavior of biological neural networks and processes distributed and parallel information (33). XGBoost is an optimized distributed gradient boosting algorithm that uses a second-order Taylor expansion to approximate the loss function, which efficiently avoids overfitting problems by adding a regularization term to the objective function, providing excellent predictions by transforming a set of weak learners into strong learners (34).

In this study, we randomly divided the data into five equal subsets. Four subsets were used to train the model and were then validated in the remaining subsets. In this process, hyperparameter adjustment was performed for the higher area under the receiver operating characteristic curve (AU-ROC), which could evaluate the prediction ability of the model. The hyperparameters were determined using a grid search, which can be tuned and scored in a loop. Changing the subset ratio to display the learning curve of the AU-ROC model helps prevent overfitting and underfitting. After adjusting the hyperparameters, the final model of the entire training set was obtained, and then the model was evaluated on the test set. The LR, RF, SVM, and MLP models were implemented in Python (version 3.7.9) using the scikit-learn package. The XGBoost model was implemented using the Python XGBoost package.

### 2.6 Hyperparameters Adjustment of Microvascular Invasion-Predicting Model

Hyperparameters were fully optimized since the training log-loss decreased as the number of trees increased; when the test log-loss was <0.693(-log0.5 = 0.693, the test log-loss of blind guess was 0.693; a lower log-loss means a better prediction) or only slightly larger than the training log-loss, the hyperparameters were fully adjusted. We ran 100 bootstrap iterations to determine the number of trees in the final model, as recommended in previous literature. Based on the grid search, the hyperparameters used in XGBoost were set as follows: learning rate = 0.13, minimum child weight = 1, maximum tree depth = 6, and number of rounds = 100. The hyperparameters used in the other models are presented in **Supplemental Table 2**.

### 2.7 Model Performance Evaluation

To evaluate the prediction performance of the various ML models, the AUC was measured and compared. We also used precision recall curve (PRC) to measure the number of positive examples that were correctly classified, which better reflecting the predictive performance when an imbalance between the groups exists. The confusion matrix was used to visually describe the accuracy of XGBoost in identifying the MVI and NMVI statuses, including true positive (TP), false positive (FP), true negative (TN), and false negative (FN).


Accuracy=(TP+TN)/(TP+FN+TN+FP)

Specificity=TN/(TN+FP)

### 2.8 Interpretation of the Model by the Shapley Additive exPlanation

It is critical to correctly interpret the prediction model. Thus, the SHAP algorithm (35), a game-theoretic approach to explain the output of any ML model, was employed to obtain accurate attribution values for each feature within the prediction model. The SHAP value can be considered a quantified contribution. We can easily determine the contribution of all features and which contribution is the most.

## 3 Results

### 3.1 Basic Characteristics

The characteristics of the 2160 patients with HCC are summarized in **Table 1**; 575 (27%) had MVI and 1585 (73%) had NMVI. The mean age of the patients was 53.2 years. The Han ethnic group accounted for 94.7% of the population. HBV positivity was found in 1773 (82%) cases. Hepatitis C virus positivity was observed in 23 cases (1.1%). HCC with cirrhosis was observed in 911 (42.2%) patients. We randomly divided these patients and allocated 80% of them to the training set and the remaining 20% to the test set. For all variables, the differences between the training and test sets were not significant. Details are presented in **Supplemental Table 3**.

**Table 1:** The participant baseline characteristics data.

Variables	Total (N=2160)	NMVI (N=1585)	MVI (N=575)	*P* Value
**Age (years)**	53.2 (11.6)	53.6 (11.4)	52.0 (12.0)	0.004
**Gender, n (%)**				0.239
** Male**	1813 (83.9)	1321 (83.3)	492 (85.6)	
** Female**	347 (16.1)	264 (16.7)	83 (14.4)	
** Height, mean (SD)**	165.1 (7.0)	165.1 (7.0)	165.2 (7.0)	0.857
** Weight, mean (SD)**	63.2 (10.2)	63.5 (10.40)	62.5 (9.6)	0.051
** BMI, mean (SD)**	23.2 (3.1)	23.3 (3.2)	22.9 (2.9)	0.008
**Nation, n (%)**				0.313
** Tibetan**	76 (3.5)	50 (3.2)	26 (4.5)	
** Han**	2046 (94.7)	1507 (95.1)	539 (93.7)	
** Others**	38 (1.8)	28 (1.8)	10 (1.7)	
**HBV, n (%)**				0.113
** Yes**	1773 (82)	1314 (82.9)	459 (79.8)	
** No**	387 (18)	271 (17.1)	116 (20.2)	
**HCV, n (%)**				0.768
** Yes**	23 (1.1)	18 (1.1)	5 (0.9)	
** No**	2137 (98.9)	1567 (98.9)	570 (99.1)	
**cirrhosis, n (%)**				0.114
** Yes**	911 (42.2)	685 (43.2)	226 (39.3)	
** No**	1249 (57.8)	900 (56.8)	349 (60.7)	

### 3.2 Clinical Characteristic Differences Between the Study Groups

The preoperative clinical characteristics of all the patients are shown in **Table 2**. Overall, in terms of imaging features, the MVI group had a larger maximum tumor diameter than the NMVI group (7.1 cm ± 3.7 cm versus [*vs.*] 4.9 cm ± 3.1 cm, *p* < 0.001). The occurrence frequency of satellite nodules (19.5% *vs.* 7.4%, *p* < 0.001) and intra-tumoral artery (29.1% *vs.* 14.4%, *p* < 0.001) were higher in the MVI group than in the NMVI group. Regarding laboratory examinations, the MVI group had a higher PLT count (158.9 × 10^9^/L ± 77.9 × 10^9^/L *vs.* 136.2 × 10^9^/L ± 67.6 × 10^9^/L, *p* < 0.001), aspartate aminotransferase (AST) level (55.3 IU/L ± 49.1 IU/L *vs.* 43.7 IU/L ± 38.3 IU/L, *p* < 0.001), AST/alanine aminotransferase (ALT) ratio (1.3 ± 0.9 *vs.* 1.1 ± 0.5, *p* < 0.001), γ-glutamyl transferase (GGT) level (116.7 IU/L ± 127.2 IU/L *vs.* 85.6 IU/L ± 122.8 IU/L, *p* < 0.001), lactate dehydrogenase level (215.2 IU/L ± 118.9 IU/L *vs.* 186.8 IU/L ± 69.5 IU/L, *p* < 0.001), hydroxybutyrate dehydrogenase (HBDH) level (160.6 IU/L ± 83.1 IU/L *vs.* 146.1 IU/L ± 54.7 IU/L, *p* < 0.001), AFP level >400 ng/mL (50.0% *vs.* 28.9%, *p* < 0.001), and PIVKA-II level (11905 mAU/mL ± 21680.9 mAU/mL *vs.* 3009.1 mAU/mL ± 9716.8 mAU/mL, *p* < 0.001) than the NMVI group. Moreover, the MVI group had more abnormal imaging and laboratory examination results than the NMVI group (**Supplemental Table 1**).

**Table 2:** The clinical characteristic differences between MVI and NMVI group.

Variables	Total (N=2160)	NMVI (N=1585)	MVI (N=575)	*P* Value
**Imaging result**				
**Satellite nodules, n (%)**				<0.001
** Yes**	230 (10.6)	118 (7.4)	112 (19.5)	
** No**	1930 (89.4)	1467 (92.6)	463 (80.5)	
**Maximum image diameter, mean (SD)**	5.5 (3.4)	4.9 (3.1)	7.1 (3.7)	<0.001
**Intratumorally artery, n (%)**				<0.001
** Yes**	343 (18.3)	198 (14.4)	145 (29.1)	
** No**	1527 (81.7)	1173 (85.6)	354 (70.9)	
**Laboratory result**				
** PLT, mean (SD)**	142.2 (71.2)	136.2 (67.6)	158.9 (77.9)	<0.001
** NEUT%, mean (SD)**	60.2 (10.0)	59.6 (10.1)	61.7 (9.7)	<0.001
** LYMPH%, mean (SD)**	28.9 (8.8)	29.4 (8.8)	27.5 (8.7)	<0.001
** NLR, mean (SD)**	2.5 (1.5)	2.4 (1.4)	2.7 (1.7)	<0.001
** FIB, mean (SD)**	2.7 (1.0)	2.6 (0.9)	2.9 (1.0)	<0.001
** AST, mean (SD)**	46.8 (41.8)	43.7 (38.3)	55.3 (49.1)	<0.001
** A/A, mean (SD)**	1.2 (0.7)	1.1 (0.5)	1.3 (0.9)	<0.001
** ALP, mean (SD)**	103.9 (62.2)	100.4 (61.1)	113.7 (64.3)	<0.001
** GGT, mean (SD)**	93.9 (124.8)	85.6 (122.8)	116.7 (127.2)	<0.001
** LDL-C, mean (SD)**	2.4 (0.8)	2.4 (0.7)	2.5 (0.9)	<0.001
** LDH, mean (SD)**	194.4 (86.4)	186.8 (69.5)	215.2 (118.9)	<0.001
** HBDH, mean (SD)**	150.0 (63.8)	146.1 (54.7)	160.6 (83.1)	<0.001
**HBV DNA, n (%)**				<0.001
** Negative**	616 (38.2)	484 (41.8)	132 (29.2)	
** Positive**	995 (61.8)	675 (58.2)	320 (70.8)	
** HBV DNA Log, mean (SD)**	3.2 (2.1)	3.0 (2.1)	3.5 (2.0)	<0.001
**AFP, n (%)**				<0.001
** <400**	1404 (65.5)	1118 (71.1)	286 (50.0)	
** >400**	740 (34.5)	454 (28.9)	286 (50.0)	
**CA-125, n (%)**				<0.001
** <35**	1270 (78.1)	969 (81.4)	301 (69.0)	
** >35**	357 (21.9)	222 (18.6)	135 (31.0)	
**PIVKA-II, n (%)**	5597.0 (14822.6)	3009.1 (9716.8)	11905.3 (21680.9)	<0.001
** <2000**	628 (29.1)	524 (33.1)	104 (18.1)	
** >2000**	503 (23.3)	278 (17.5)	225 (39.1)	

### 3.3 Development and Validation of the MVI-Predicting Model

#### 3.3.1 Development of the MVI-Predicting Model

**Figure 2:**
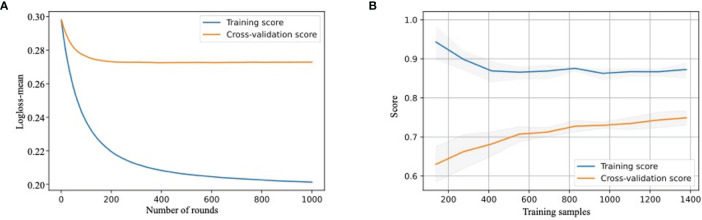
Development and validation of MVI-prediction model **(A)** The training process of XGBoost model. Train-log-loss-mean value for the training datasets is shown in the vertical axis. The horizontal axis represents the number of times iterative cross-validation. **(B)** The learning curve of the score of training cohort and testing cohort. The score for training and test cohorts is shown in the vertical axis. The horizontal axis represents the number of samples trained.

All models were parameterized with these hyperparameters, and bootstrap validation training log-loss decreased as the number of integration trees increased. The bootstrap validation testing log-loss was <0.693, which was only slightly higher than the training log-loss when the number of rounds increased. Here, we only show the training curve of the XGBoost model as an example, which indicated a good fitting (**Figure 2A**). When the sample size reached 200 rounds, the log-loss of the training and test sets gradually tended to be stable, indicating that the model was well turned.

The learning curve showed that as the score of the training cohort decreased, the score of the validation cohort increased with an increase in training samples. We also used the XGBoost model for demonstration (**Figure 2B**). This revealed that as the sample size increased, the model had not been overfitted or underfitted, indicating a robust predictive performance.

#### 3.3.2 Model Performance

**Figure 3:**
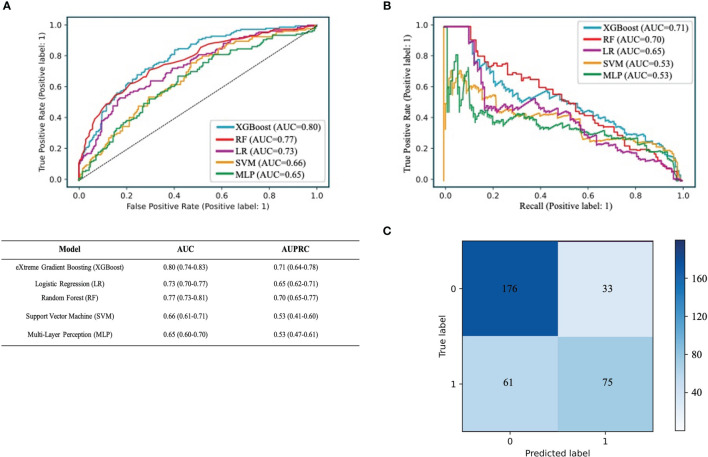
Performance of the predictive models. **(A)** The ROC curve analysis of various prediction model. **(B)** The PRC curve of different models. The confusion matrix of XGBoost model in the validation cohort. **(C)** The confusion matrix of XGBoost model. The confusion matrix was composed of the True negative in the first quadrant, the false negative samples in the second quadrant, the true positive example in the third quadrant and the false positive example in the fourth quadrant.

We used RF, SVM, LR, XGBoost, and MLP algorithms to construct and optimize the MVI prediction model, and found that the XGBoost model achieved the highest AUC (0.8, 95% confidence interval [CI]: 0.74–0.83) (**Figure 3A**), followed by the RF (0.77, 95% CI: 0.73–0.81), LR (0.73, 95% CI: 0.70–0.77), SVM (0.66, 95% CI: 0.61–0.71), and MLP models (0.65, 95% CI: 0.60–0.70). Since the positive and negative samples were highly skewed datasets in our study, we used PRC to reflect the performance of the classifier more effectively. The area under the precision recall curve (AUPRC) value of the XGBoost model was much higher (0.71, 95% CI: 0.64–0.78) than that of the other models (**Figure 3B**). Other algorithms showed the following AUPRC values: RF model, 0.7, 95% CI: 0.65–0.77; LR model, 0.65, 95% CI: 0.62–0.71; SVM model, 0.53, 95% CI: 0.41–0.60; and MLP model, 0.53, 95% CI: 0.47–0.61. Additionally, the confusion matrix showed that the accuracy and specificity of the XGBoost model were 73% and 84%, respectively (**Figure 3C**).

### 3.4 Model Interpretation

**Figure 4:**
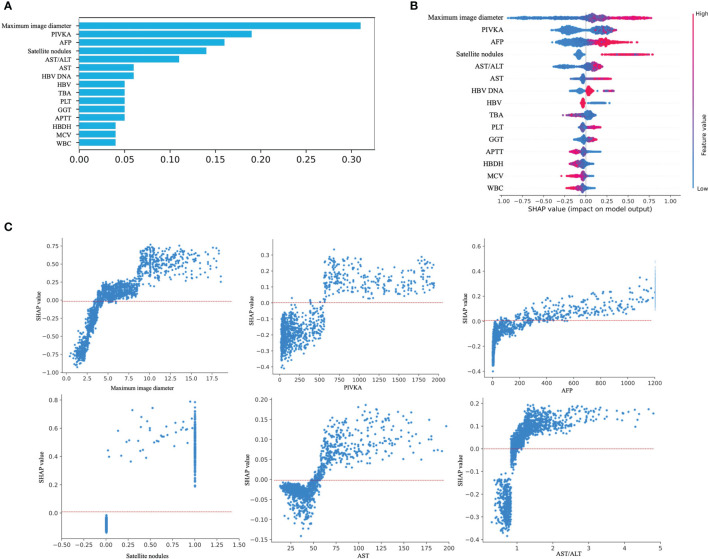
Model interpretation. **(A)** Feature importance matrix plot derived from XGBoost model. **(B)** SHAP summary plot of the XGBoost model. The higher the SHAP value for each feature, the higher risk of MVI development. A dot represents each feature contribution for each patient in the model. Red indicates a high SHAP value, blue indicates a low SHAP value. **(C)** SHAP dependance plot of XGBoost model. The SHAP dependance plot represents the contribution of each feature that we care about to the output of the XGBoost model. If the SHAP value of the feature we care about is exceeds zero, the higher the risk of MVI will be.

The feature importance matrix plot sorted the most important variables, revealing the contribution of each variable to MVI versus NMVI. The top six factors associated with MVI were the maximum image diameter, PIVKA-II level, AFP level, satellite nodules, AST/ALT ratio, and AST level (**Figure 4A**). To illustrate the influence of each feature on model prediction, we drafted the SHAP value summary chart, and only the top 15 variables of the model are shown (**Figure 4B**). The chart shows the correlation between the high or low SHAP values and the prediction model. We observed that the red dots, which represent the high values of the maximum image diameter, AFP level, satellite nodules, AST/ALT ratio, and AST level, appeared more on the side of the higher probability risk of MVI. This indicates that the SHAP values of these indicators were positively correlated with the possibility of the occurrence of MVI. The red dots representing the high values of the PIVKA-II level were covered by blue dots, indicating lower values of the PIVKA-II level. This indicated that predication was affected by the extreme values, and even if the PIVKA-II values were high, some points were still blue and tended to predict the occurrence of NMVI. Furthermore, the SHAP values of the above features are displayed, which show a clear distinction between MVI and NMVI. The cutoff values of the maximum image diameter, PIVKA-II level, AFP level, satellite nodules, AST/ALT ratio, and AST level were 5 cm, 500 mAU/mL, 200 ng/mL, one nodule, 1, and 50 U/L, respectively (**Figure 4C**). By integrating the SHAP value summary chart and the SHAP scatter plot, the sensitivity of PIVKA-II was found to be good, while the specificity of PIVKA-II was not. Other indicators showed good specificity and sensitivity.

### 3.5 Online Calculator Based on the Extreme Gradient Boosting Microvascular Invasion Prediction Model

We established a website based on the XGBoost model to predict the risk of MVI (https://260147169.github.io/MVI-calculator/MVI-calculator.html). We only needed to fill in the corresponding parameters of each indicator, including the maximum image diameter, PIVKA-II level, AFP level, satellite nodules, AST/ALT ratio, AST levels, HBV, total bile acid level, PLT count, GGT level, APTT, HBDH level, mean red blood cell volume, and white blood cell count. The online calculator automatically and promptly converted the MVI risk score (**Supplemental Figure 1**).

## 4 Discussion

In this study, we developed an XGBoost model for the preoperative prediction of MVI based on the EHR information of 2160 patients with HCC at the West China Hospital, and it exhibited the best AUC in the validation set compared with the other ML algorithms and showed good interpretability, as well as the importance of MVI-related factors. Furthermore, we built an online calculator based on this model to make prediction of MVI more practical. Overall, we provide a valuable and reliable preoperative MVI prediction model, which may be effective in optimizing surgical treatment and further improving the survival of patients with HCC.

Notably, the lack of specific and effective preoperative indicators is one of the bottlenecks in the diagnosis of MVI. In this retrospective cohort study, we developed an XGBoost model using 88 objective preoperative demographic, imaging, and laboratory indicators to predict the possibility of MVI. AUC analysis alone is often insufficient for comparing predictive models, particularly in an imbalanced dataset. Therefore, we used both the AUC and AUPRC to evaluate the performance of the five ML methods. Compared with other models, the XGBoost model exhibited a better performance with an AUC of 0.8 and an AUPRC of 0.71.

Several ML methods have been developed to predict the risk of MVI. Some methods were based on radiomics or included only a few vital tumor biomarkers to build ML algorithms to predict MVI (36). Dong et al. (37) established a radiomic algorithm to make preoperative predictions of MVI based on grayscale ultrasonograms. The radiomic signatures based on the features of the gross tumor region (GTR), peri-tumoral region, and gross peritumoral region (GPTR) showed AUCs of 0.708, 0.710, and 0.726, respectively. After incorporating some important tumor biomarkers, the AUC of the GPTR radiomic signature was 0.744, and the AUC of the GTR radiomic signature was 0.806. This might ignore useful information, especially some serum biomarkers. It is worth noting that our model was built based on three multidimensional preoperative indicators, including patient clinical characteristics, imaging examination features, and laboratory examination results, without invasive examination. Compared with the MVI prediction models using radiomics as the only predictor (25, 38), the potential significance of this model with multivariable predictors was that we could predict the possibility of MVI using routine clinical information before surgery.

Our work also has the advantages of convenient data collection, ready availability, and objectivity, which are suitable for use in the evaluation of MVI in most clinical situations. Remarkably, all the variables in our model have a short detection time, which can help clinicians quickly obtain reference diagnostic information for patients with no immediate access to pathological diagnosis. Additionally, previous studies constructed an MVI prediction model based on a small sample size (25). Studies have shown that the models with a large sample size have higher robustness than those with a small sample size (26); the sample size of our study was much bigger than that of previous studies as far as we know. Furthermore, our model showed consistent performance between the observed and predicted MVI risks by SHAP values, implying the interpretation and robustness of the model.

The importance ranking of the correlation between the top 15 conventional imaging and laboratory variables with the occurrence of MVI was identified through XGBoost model learning. Specifically, the maximum image diameter, PIVKA-II level, AFP level, satellite nodules, AST/ALT ratio, and AST level showed the top six significant contributions to the prediction of MVI, whether using the importance matrix plot or SHAP summary plot of the XGBoost model. Among them, the maximum image diameter was ranked first. In addition, satellite nodules, one of the imaging indicators, were in the top six importance rankings. Roayaie et al. defined satellite nodules as tumors ≤2 cm in size located ≤2 cm from the main tumor (39). In our SHAP scatter plot of satellite nodules, most of the scatter point values were 1. Our SHAP scatter plot of the maximum image diameter showed that the cutoff value was 5 cm. Recently, Zhang et al. (40) demonstrated that the maximum image diameter and emergence of satellite nodules aggravated the MVI of HCC. Their studies set the cutoff value of the maximum image diameter to 5 cm by referring to different guidelines. They also indicated that the presence of satellite nodules might be a risk factor for predicting the occurrence of MVI. This finding is consistent with our results.

In addition to imaging indicators, laboratory indicators, such as the PIVKA-II level, AFP level, AST/ALT ratio, and AST level, also appeared in the top six SHAP value rankings. This observation was consistent with that of a previous report in which high levels of AFP and PIVKA-II were found to be closely related to MVI (41, 42). Meanwhile, in the SHAP scatter plot, the cutoff of our PIVKA-II level was >500 mAU/L. There were some patients with NMVI even though PIVKA-II values were high; our data showed that the specificity of the PIVKA-II level was relatively poor, and we thought that the cutoff value should be improved. In previous studies, Fumitoshi et al. (43) performed a univariate analysis of 167 patients, revealing that a PIVKA-II level ≥150 mAU/mL on preoperative examination was a high risk factor for MVI. In a study by Pote et al., a PIVKA-II level >90 mAU/mL was an independent predictor of MVI (10). However, this finding is in line with the clinical expectation that a larger PIVKA-II value is more strongly correlated with HCC.

In our research, a consistent tendency was found in that the SHAP values with scattered points above 0 was almost always >200 ng/mL on the SHAP plot of the AFP level. You et al. (44) analyzed 215 patients who underwent liver resection using univariate and multivariate analyses, and showed that an AFP cutoff level of 400 ng/mL was an independent risk factor associated with MVI. The cutoff value of the AFP level in the present study was slightly smaller than that reported in previous studies.

As indicators of impaired liver function, the AST/ALT ratio and AST level were also ranked in importance. In our study, patients with a higher AST/alkaline phosphatase (ALP) ratio and AST level were more likely to develop MVI than those with a lower AST/ALP ratio and AST level, and the cutoff value of the AST/ALT ratio was almost higher than 1 on the SHAP scatter plot. The SHAP-scattered points of serum AST levels were almost greater than 50 U/L. A previous study reported that ALT is mainly present in the cytoplasm of hepatocytes; whereas, AST mainly exists in the mitochondria of hepatocytes, and an increase in its level indicates that hepatocytes have damaged organelles. Therefore, an increased AST/ALT ratio could generally be considered indicative of the deterioration of liver cell damage in patients with cirrhosis and HCC (45). Yang et al. (46). reported that the AST/ALT ratio is often >1 due to the invasion of hepatic carcinoma cells. Dong et al. revealed that an AST level >40 U/L was an independent factor for overall survival in HCC (47). Our SHAP scatter plot confirmed this. Overall, the variables selected for our prediction model were the most clinically common, readily available, and short-duration imaging and laboratory indicators, and they showed good interpretability and consistency with clinical experience, further proving the reliability of the model. This also shows the possibility that our model can be applied to countries and regions with relatively limited medical resources.

The strengths of this study are as follows. First, we used a large dataset to build an ML model for the preoperative prediction of MVI. This could contribute to improving the effective training and rational explanation of the prediction model so that the model was closer to the real situation of the prediction power. Second, we used multiple dimensional indicators to build the prediction model, thus improving its performance. All predictors have the advantages of convenient data collection, ready availability, and objectivity. Third, we used a variety of ML algorithms to select the optimal model that best fits this dataset. Finally, we transformed the model into a visual software based on the selected 15 common clinical indicators that facilitate rapid detection. Thus, the prediction model can be easily applied to countries and regions with relatively limited medical resources.

Despite these advantages, our study also has some limitations. First, this was a retrospective study, and the findings need to be validated in prospective studies. Second, our model was developed based on a single center, so its generalizability needs to be verified in multiple centers. Third, we only constructed a preoperative MVI prediction model; therefore, the clinical benefit of precise surgical choice based on the model needs to be evaluated in the future.

## 5 Conclusion

In conclusion, our study constructed and validated different ML algorithm models for the preoperative diagnosis of MVI by utilizing preoperative readily available, short-duration, and general noninvasive preoperative indicators. In the final model, we chose the XGBoost algorithm because it had the best performance in predicting MVI. The maximum image diameter, PIVKA-II level, AFP level, satellite nodules, AST/ALT ratio, and AST level were found to be important for predicting the occurrence of MVI. Further, development of the MVI risk-scoring web-calculator based on this model is convenient for clinical application. Meanwhile, the developed model is helpful in preoperatively predicting MVI and assists clinicians in conveniently determining the optimal therapeutic remedy and ameliorating the prognosis of patients with HCC.

## Data Availability Statement

The datasets presented in this study can be found in online repositories. The names of the repository/repositories and accession number(s) can be found in the article/**Supplementary Material**.

## Ethics Statement

The studies involving human participants were reviewed and approved by This study was approved by the Institutional Ethics Committee of the West China Hospital, Sichuan University [No. 2019 (203)]. The patients/participants provided their written informed consent to participate in this study.

## Author Contributions

Research conception: BY, JZ, and WL. Data processing: LZ and WL. Drafting of the manuscript: WL, LZ, ZX, LY, and LB. Data acquisition: HZ and JZ. Revision of the manuscript: BY and JZ. All authors contributed to the article and approved the submitted version.

## Funding

This work was supported by the National Natural Science Foundation of China (NO. 81873979), the Project of Sichuan Provincial Department of Science and Technology (NO. 2020YFS0214, 2020YJ0106).

## Conflict of Interest

The authors declare that the research was conducted in the absence of any commercial or financial relationships that could be construed as a potential conflict of interest.

## Publisher’s Note

All claims expressed in this article are solely those of the authors and do not necessarily represent those of their affiliated organizations, or those of the publisher, the editors and the reviewers. Any product that may be evaluated in this article, or claim that may be made by its manufacturer, is not guaranteed or endorsed by the publisher.

## Glossary

**Table:**

AFP	α-fetoprotein
AI	artificial intelligence
ALB	albumin
ALP	alkaline phosphatase
ALP/GGT (A/G)	alkaline phosphatase/γ-glutamyl transferase
ALT	alanine aminotransferase
APTT	activated partial thromboplastin time
AST	aspartate aminotransferase
AST/ALT (A/A)	aspartate aminotransferase/alanine aminotransferase
AUC	the area under the receiver operating characteristic curve
BASO%	basophil percentage
BMI	body mass index
CA-125	carbohydrate antigen 125
CA19-9	carbohydrate antigen
CEA	carcinoembryonic antigen
CHOL	cholesterol
CK	creatine kinase
CREA	creatinine
Cys-C	cystatin C
DBIL	Direct bilirubin
eGFR	estimated glomerular filtration rate
EHR	electronic health record
EO%	Percentage of eosinophils
FIB	fibrinogen
FN	False Negative
FP	False Positive
GGT	γ-glutamyl transferase
GGTP	gamma glutamyl transpeptidase
GLB	globulin
GLU	glucose
Hb	hemoglobin
HBcAb	hepatitis B core antibody
HBDH	hydroxybutyrate dehydrogenase
HBeAb	hepatitis B e antibody
HBeAg	hepatitis B e antigen
HBsAb	hepatitis B s antibody
HBsAg	hepatitis B s antigen
HBV	hepatitis B virus
HBV DNA	hepatitis B virus DNA
HCV	hepatitis C virus
HCC	hepatocellular carcinoma
Hct	hematocrit
HDL-C	high-density lipoprotein cholesterol
IBIL	indirect bilirubin
IG%	Percentage of naive granulocytes
IG |	absolute value of immature granulocytes
LDH	lactate dehydrogenase
LDL-C	low-density lipoprotein cholesterol
LYMPH%	percentage of lymphocytes
MCH	mean corpuscular hemoglobin
MCHC	mean corpuscular hemoglobin concentration
MCV	mean red blood cell volume
MONO%	monocyte percentage
ML	machine learning
MLP	Multi-Layer Perception
MVI	microvascular invasion
NEUT%	neutral lobulated granulocyte percentage
NLR	the neutrophilic lymphocyte ratio
PIVKA-II	protein induced by vitamin K absence or antagonist-II
PLT	platelets
PRC	precision recall curve
PT	prothrombin time
RBC	red blood cells
RDW-CV	RBC distribution width-coefficient of variation
RDW-SD	RBC distribution width-standard deviation
RF	random forest
RFA	radiofrequency ablation
RFS	relapse free survival
SHAP	Shapley Addictive explanation
SVM	support vector machine
TACE	transcatheter arterial chemoembolization
TBA	total bile acid
TBIL	total bilirubin
TN	Ture Negative
TP	True Positive
TT	thrombin time
UREA	uric acid
WBCs	white blood cells
XGBoost	extreme gradient boosting
