# Supplementary material for: A Promising Preoperative Prediction Model for Microvascular Invasion in Hepatocellular Carcinoma Based on an Extreme Gradient Boosting Algorithm
Source: Front Oncol. 2022 Mar 4;12:852736. doi: 10.3389/fonc.2022.852736 (PMC8931027; doi:10.3389/fonc.2022.852736)
Supplement: Supplementary file 1 [file DataSheet_1.docx]

Supplementary Material

# Hyperparameters adjustment of MVI-Predicting model

RF: n-estimators = 100, min-samples-split = 2, min-samples-leaf = 1, min-weight-fraction-leaf = 0, min-impurity-decrease = 0, max-depth = 7

SVM: nu = 0.5, degree = 3, coef 0 = 0, total = 0.001

MLP: hidden-layer-sizes = (10,10,10,10), alpha = 0.00001, learning-rate-init = 0.001, power-t= 0.5, max-iter = 1000, total = 0.0001

# Supplementary Figures and Tables

## Supplementary Tables

**Supplemental Table 1** **The clinical characteristic differences between MVI and NMVI group**

| Variables | Total  (N=2160) | NMVI  (N=1585) | MVI  (N=575) | *P* Value |
| --- | --- | --- | --- | --- |
| Demographic data |  |  |  |  |
| HBV-Lord, n (%) |  |  |  | <0.001 |
| Yes | 1048 (63.7) | 740 (61.0) | 308 (71.3) |  |
| No | 598 (36.3) | 474 (39.0) | 124 (28.7) |  |
| Preoperative intervention, n (%) |  |  |  | 0.945 |
| Yes | 78 (3.6) | 58 (3.7) | 20 (3.5) |  |
| No | 2082 (96.4) | 1527 (96.3) | 555 (96.5) |  |
| Imaging result |  |  |  |  |
| Single image, n (%) |  |  |  | 0.113 |
| Single | 331 (15.7) | 231 (15.0) | 100 (17.9) |  |
| Multiple | 1772 (84.3) | 1314 (85.0) | 458 (82.1) |  |
| Number of lesions, n (%) |  |  |  | 0.041 |
| Solitary | 1814 (84.0) | 1347 (85.0) | 467 (81.2) |  |
| Multiple | 346 (16.0) | 238 (15.0) | 108 (18.8) |  |
| Imaging sub focus, n (%) |  |  |  | 0.001 |
| Yes | 63 (2.9) | 34 (2.1) | 29 (5.0) |  |
| No | 2097 (97.1) | 1551 (97.9) | 546 (95.0) |  |
| Portal hypertension, n (%) |  |  |  | 0.577 |
| Yes | 1244 (57.6) | 919 (58.0) | 325 (56.5) |  |
| No | 916 (42.4) | 666 (42.0) | 250 (43.5) |  |
| Imaging macrovascular invasion, n (%) |  |  |  | <0.001 |
| Yes | 56 (2.6) | 22 (1.4) | 34 (5.9) |  |
| No | 2104 (97.4) | 1563 (98.6) | 541 (94.1) |  |
| Imaging sub focus, n (%) |  |  |  |  |
| Yes | 63 (2.9) | 34 (2.1) | 29 (5.0) | 0.001 |
| No | 2097 (97.1) | 1551 (97.9) | 546 (95.0) |  |
| Tumor boundary, n (%) |  |  |  | 0.111 |
| Smooth | 418 (29.8) | 319 (31.1) | 99 (26.5) |  |
| Not smooth | 983 (70.2) | 708 (68.9) | 275 (73.5) |  |
| Imaging examination items |  |  |  | 0.053 |
| CT | 1467 (69.4) | 1057 (68.1) | 410 (73.2) |  |
| MRI | 403 (19.1) | 314 (20.2) | 89 (15.9) |  |
| Ultrasonic | 243 (11.5) | 182 (11.7) | 61 (10.9) |  |
| Laboratory result |  |  |  |  |
| IG%, mean (SD) | 0.3 (0.3) | 0.3 (0.2) | 0.4 (0.5) | 0.001 |
| \|IG\|, mean (SD) | 0.1 (0.7) | 0.1 (0.5) | 0.2 (0.9) | 0.025 |
| RBC, mean (SD) | 4.6 (0.6) | 4.6 (0.6) | 4.7 (0.7) | 0.014 |
| RBC, n (%) |  |  |  | 0.001 |
| Decrease | 477 (22.2) | 353 (22.4) | 124 (21.6) |  |
| Normal | 1575 (73.3) | 1165 (74.1) | 410 (71.3) |  |
| Increase | 96 (4.5) | 55 (3.5) | 41 (7.1) |  |
| Hb, mean (SD) | 141.9 (19.0) | 141.8 (18.1) | 142.0 (21.3) | 0.802 |
| Hb1, n (%) |  |  |  | <0.001 |
| Decrease | 344 (16.0) | 240 (15.3) | 104 (18.1) |  |
| Normal | 1732 (80.6) | 1296 (82.4) | 436 (75.8) |  |
| Increase | 72 (3.4) | 37 (2.4) | 35 (6.1) |  |
| Hct, mean (SD) | 0.4 (0.1) | 0.4 (0.1) | 0.4 (0.1) | 0.356 |
| MCV, mean (SD) | 93.2 (6.7) | 93.5 (6.5) | 92.5 (6.9) | 0.002 |
| MCH, mean (SD) | 30.7 (2.6) | 30.8 (2.6) | 30.4 (2.8) | 0.001 |
| MCHC, mean (SD) | 329.1 (12.0) | 329.5 (11.6) | 328.0 (13.0) | 0.015 |
| RDW-CV, mean (SD) | 13.6 (1.4) | 13.6 (1.3) | 13.7 (1.5) | 0.102 |
| RDW-SD, mean (SD) | 46.1 (4.5) | 46.2 (4.5) | 45.9 (4.5) | 0.269 |
| PLT, n (%) |  |  |  | <0.001 |
| Decrease | 638 (29.7) | 498 (31.7) | 140 (24.3) |  |
| Normal | 1439 (67.0) | 1039 (66.1) | 400 (69.6) |  |
| Increase | 71 (3.3) | 36 (2.3) | 35 (6.1) |  |
| WBC, mean (SD) | 5.5 (1.9) | 5.4 (1.8) | 5.6 (2.0) | 0.044 |
| WBC, n (%) |  |  |  |  |
| Decrease | 257 (12.0) | 197 (12.5) | 60 (10.4) | 0.225 |
| Normal | 1808 (84.2) | 1320 (83.9) | 488 (84.9) |  |
| Increase | 83 (3.9) | 56 (3.6) | 27 (4.7) |  |
| MONO%, mean (SD) | 7.7 (2.0) | 7.6 (2.0) | 7.7 (2.1) | 0.429 |
| EO%, mean (SD) | 2.7 (2.4) | 2.8 (2.5) | 2.5 (2.1) | 0.005 |
| BASO%, mean (SD) | 0.5 (0.3) | 0.5 (0.3) | 0.5 (0.3) | 0.065 |
| PT, mean (SD) | 12.3 (2.7) | 12.3 (3.1) | 12.3 (1.2) | 0.722 |
| APTT, mean (SD) | 30.1 (4.5) | 30.1 (4.2) | 30.1 (5.1) | 0.763 |
| TT, mean (SD) | 20.3 (1.7) | 20.4 (1.7) | 20.2 (1.7) | 0.096 |
| TBIL, mean (SD) | 15.8 (14.0) | 15.5 (12.1) | 16.6 (18.0) | 0.192 |
| DBIL, mean (SD) | 6.5 (11.2) | 6.2 (9.4) | 7.3 (15.0) | 0.094 |
| IBIL, mean (SD) | 9.3 (4.9) | 9.3 (4.8) | 9.3 (5.2) | 0.813 |
| ALT, mean (SD) | 45.8 (45.4) | 44.9 (44.5) | 48.3 (47.8) | 0.141 |
| TP, mean (SD) | 70.9 (6.0) | 70.9 (5.8) | 70.9 (6.3) | 0.999 |
| ALB, mean (SD) | 42.4 (4.3) | 42.5 (4.2) | 41.9 (4.6) | 0.007 |
| GLB, mean (SD) | 28.5 (4.9) | 28.4 (4.7) | 29.0 (5.4) | 0.020 |
| A/G, mean (SD) | 1.5 (0.3) | 1.5 (0.3) | 1.5 (0.3) | 0.009 |
| GLU, mean (SD) | 5.4 (1.8) | 5.4 (1.6) | 5.4 (2.1) | 0.750 |
| UREA, mean (SD) | 5.4 (1.6) | 5.5 (1.6) | 5.4 (1.7) | 0.103 |
| CREA, mean (SD) | 71.0 (15.0) | 71.1 (15.0) | 70.7 (15.0) | 0.665 |
| eGFR, mean (SD) | 98.0 (14.9) | 97.3 (15.0) | 99.5 (15.4) | 0.044 |
| Cys-C, mean (SD) | 1.0 (0.2) | 1.0 (0.2) | 1.0 (0.2) | 0.181 |
| URIC, mean (SD) | 328.9 (80.0) | 327.5 (79.8) | 332.5 (80.6) | 0.203 |
| TG, mean (SD) | 1.1 (0.7) | 1.1 (0.7) | 1.1 (0.6) | 0.041 |
| CHOL, mean (SD) | 4.2 (1.0) | 4.2 (0.9) | 4.3 (1.1) | 0.007 |
| HDL-C, mean (SD) | 1.3 (0.4) | 1.3 (0.4) | 1.2 (0.4) | 0.001 |
| CK, mean (SD) | 95.0 (140.5) | 98.2 (158.1) | 86.5 (71.8) | 0.021 |
| TBA, mean (SD) | 13.7 (23.4) | 13.9 (24.2) | 13.1 (21.4) | 0.619 |
| CA19-9, mean (SD) | 29.3 (65.8) | 28.2 (61.8) | 32.4 (75.8) | 0.235 |
| CEA, mean (SD) | 2.9 (7.0) | 2.9 (6.7) | 2.8 (7.8) | 0.824 |
| CA-125, mean (SD) | 23.6 (111.9) | 20.3 (51.7) | 32.6 (198.5) | 0.203 |
| AFP, mean (SD) | 1915.3 (17384.4) | 907.0 (5735.3) | 4686.5 (32144.2) | 0.005 |
| PIVKA-II, mean (%) | 5597.0 (14822.6) | 3009.1 (9716.8) | 11905.3 (21680.9) | <0.001 |
| HBsAg, n (%) |  |  |  | 0.969 |
| Negative | 367 (17.1) | 268 (17.0) | 99 (17.2) |  |
| Positive | 1782 (82.9) | 1306 (83.0) | 476 (82.8) |  |
| HBsAb, n (%) |  |  |  | 0.811 |
| Negative | 1784 (83.0) | 1309 (83.2) | 475 (82.6) |  |
| Positive | 365 (17.0) | 265 (16.8) | 100 (17.4) |  |
| HBeAg, n (%) |  |  |  | 0.043 |
| Negative | 1311 (61.0) | 981 (62.3) | 330 (57.4) |  |
| Positive | 838 (39.0) | 593 (37.7) | 245 (42.6) |  |
| HBeAb, n (%) |  |  |  |  |
| Negative | 482 (22.4) | 354 (22.5) | 128 (22.3) | 0.956 |
| Positive | 1667 (77.6) | 1220 (77.5) | 447 (77.7) |  |
| HBeAg, n (%) |  |  |  | 0.043 |
| Negative | 1311 (61.0) | 981 (62.3) | 330 (57.4) |  |
| Positive | 838 (39.0) | 593 (37.7) | 245 (42.6) |  |
| HBcAb, n (%) |  |  |  |  |
| Negative | 50 (2.3) | 37 (2.4) | 13 (2.3) | 0.969 |
| Positive | 2099 (97.7) | 1537 (97.6) | 562 (97.7) |  |

**Supplemental Table 2** **Multivariable logistic regression model of MVI with stepwise variable selection based on preoperative data**

| **Variables** | **OR (95% CI)** | ***P V*alue** |
| --- | --- | --- |
| **HBV** | 0.49 [0.40, 0.58] | < 0.001 |
| **Satellite nodules** | 1.90 [1.66, 2.17] | < 0.001 |
| **Imaging macrovascular invasion** | 2.35 [1.79, 3.09] | 0.001 |
| **Maximum Image diameter** | 1.07 [1.05, 1.09] | < 0.001 |
| **Gender** | 1.51 [1.32, 1.73] | 0.003 |
| **IG%** | 1.54 [1.28, 1.86] | 0.021 |
| **MCV** | 0.99 [0.98, 0.99] | 0.039 |
| **WBC** | 0.92 [0.90, 0.95] | 0.003 |
| **NEUT%** | 1.05 [1.03, 1.07] | 0.008 |
| **DBIL** | 1.03 [1.01, 1.04] | 0.045 |
| **A/A** | 1.31 [1.19, 1.44] | 0.005 |
| **LYMPH%** | 1.05 [1.03, 1.07] | 0.015 |
| **HBV DNA LOG** | 1.08 [1.06, 1.11] | 0.001 |
| **AFP** | 1.46 [1.33, 1.60] | < 0.001 |
| **HBsAg** | 1.56 [1.28, 1.90] | 0.025 |
| **HBeAg** | 1.39 [1.22, 1.58] | 0.012 |
| **PIVKA-II** | 1.31 [1.19, 1.45] | 0.005 |

**Supplemental Table 3 The clinical characteristic differences between training set and testing set**

| Variables | | Total  (N=1724) | Training Set  (N=1379) | Testing Set  (N=345) | *P* Value |
| --- | --- | --- | --- | --- | --- |
| Demographic data | |  |  |  |  |
| Age (years) | | 52.8 (11.6) | 52.7 (11.6) | 53.2 (11.4) | 0.518 |
| Gender, n (%) | |  |  |  | 0.983 |
| Male  Female | | 1451 (84.2)  273 (15.8) | 1161 (84.2)  218 (15.8) | 290 (84.1)  55 (15.9) |  |
| Height, mean (SD) | | 165.0 (6.9) | 164.9 (7.0) | 165.2 (6.6) | 0.500 |
| Weight, mean (SD) | | 62.9 (10.0) | 62.9 (10.0) | 63.1 (9.8) | 0.664 |
| BMI, mean (SD) | | 23.1 (3.0) | 23.1 (3.0) | 23.1 (3.0) | 0.830 |
| Nation, n (%) | |  |  |  | 0.313 |
| Tibetan | | 61 (3.5) | 43 (3.1) | 18 (5.2) | 0.100 |
| Han | | 1631 (94.6) | 1308 (94.9) | 323 (93.6) |  |
| Others | | 32 (1.9) | 28 (2.0) | 4 (1.2) |  |
| HBV, n (%) | |  |  |  | 0.113 |
| Yes  No | | 1773 (82.1)  387 (17.9) | 1314 (82.9)  271 (17.1) | 459 (79.8)  116 (20.2) |  |
| HBV-lord, n (%) | |  |  |  | 0.958 |
| Yes | | 1054 (61.1) | 843 (61.1) | 211 (61.2) |  |
| No | | 670 (38.9) | 536 (38.9) | 134 (38.8) |  |
| HCV, n (%) | |  |  |  | 0.753 |
| Yes | | 16 (0.9) | 14 (1.0) | 2 (0.6) |  |
| No | | 1708 (99.1) | 1365 (99.0) | 343 (99.4) |  |
| Preoperative intervention,  n (%) | |  |  |  | 0.824 |
| Yes | | 66 (3.8) | 54 (3.9) | 12 (3.5) |  |
| No | | 1658 (96.2) | 1325 (96.1) | 333 (96.5) |  |
| Imaging result | |  |  |  |  |
| cirrhosis, n (%) | |  |  |  | 0.420 |
| Yes | | 724 (42.0) | 572 (41.5) | 152 (44.1) |  |
| No | | 1000 (58.0) | 807 (58.5) | 193 (55.9) |  |
| Portal hypertension, n (%) | |  |  |  | 0.557 |
| Yes | | 1001 (58.1) | 806 (58.4) | 195 (56.5) |  |
| No | | 723 (41.9) | 573 (41.6) | 150 (43.5) |  |
| Single image, n (%) | |  |  |  |  |
| Single | | 274 (15.9) | 220 (16.0) | 54 (15.7) | 0.956 |
| Multiple | | 1450 (84.1) | 1159 (84.0) | 291 (84.3) |  |
| Number of lesions, n (%) | |  |  |  |  |
| Solitary | | 1433 (83.1) | 1147 (83.2) | 286 (82.9) | 0.966 |
| Multiple | | 291 (16.9) | 232 (16.8) | 59 (17.1) |  |
| Imaging sub focus, n (%) |  | |  |  | 0.186 |
| Yes | | 62 (3.6) | 45 (3.3) | 17 (4.9) |  |
| No | | 1662 (96.4) | 1334 (96.7) | 328 (95.1) |  |
| Image macrovascular invasion, n (%) | |  |  |  | 0.916 |
| Yes | | 54 (3.1) | 44 (3.2) | 10 (2.9) |  |
| No | | 1670 (96.9) | 1335 (96.8) | 335 (97.1) |  |
| Maximum image diameter, mean (SD) | | 5.8 (3.5) | 5.7 (3.5) | 5.9 (3.5) | 0.285 |
| Tumor boundary, n (%) | |  |  |  | 0.623 |
| Smooth | | 435 (25.2) | 352 (25.5) | 83 (24.1) |  |
| Not smooth | | 1289 (74.8) | 1027 (74.5) | 262 (75.9) |  |
| Satellite nodules, n (%) | |  |  |  | 0.845 |
| Yes | | 217 (12.6) | 172 (12.5) | 45 (13.0) |  |
| No | | 1507 (87.4) | 1207 (87.5) | 300 (87.0) |  |
| Intratumorally artery, n (%) | |  |  |  | 0.935 |
| Yes | | 340 (19.7) | 273 (19.8) | 67 (19.4) |  |
| No | | 1384 (80.3) | 1106 (80.2) | 278 (80.6) |  |
| Imaging examination items | |  |  |  |  |
| CT, n (%) | |  |  |  | 0.145 |
| Yes | | 1227 (71.2) | 970 (70.3) | 257 (74.5) |  |
| No | | 497 (28.8) | 409 (29.7) | 88 (25.5) |  |
| MRI, n (%) | |  |  |  | 0.093 |
| Yes | | 311 (18.0) | 260 (18.9) | 51 (14.8) |  |
| No | | 1413 (82.0) | 1119 (81.1) | 294 (85.2) |  |
| Ultrasonic, n (%) | |  |  |  | 0.957 |
| Yes | | 186 (10.8) | 149 (10.8) | 37 (10.7) |  |
| No | | 1538 (89.2) | 1230 (89.2) | 308 (89.3) |  |
| Laboratory result | |  |  |  |  |
| IG%, mean (SD) | | 0.3 (0.3) | 0.3 (0.4) | 0.3 (0.2) | 0.346 |
| \|IG\|, mean (SD) | | 0.1 (0.7) | 0.1 (0.7) | 0.1 (0.5) | 0.358 |
| RBC, mean (SD) | | 4.7 (0.7) | 4.6 (0.7) | 4.7 (0.7) | 0.350 |
| RBC, n (%) | |  |  |  | 0.181 |
| Decrease | | 379 (22.0) | 309 (22.4) | 70 (20.3) |  |
| Normal | | 1261 (73.1) | 1009 (73.2) | 252 (73.0) |  |
| Increase | | 84 (4.9) | 61 (4.4) | 23 (6.7) |  |
| Hb, mean (SD) | | 141.9 (19.3) | 141.9 (19.1) | 141.8 (19.7) | 0.898 |
| Hb, n (%) | |  |  |  | 0.531 |
| Decrease | | 275 (16.0) | 223 (16.2) | 52 (15.1) |  |
| Normal | | 1381 (80.1) | 1105 (80.1) | 276 (80.0) |  |
| Increase | | 68 (3.9) | 51 (3.7) | 17 (4.9) |  |
| Hct, mean (SD) | | 0.4 (0.1) | 0.4 (0.1) | 0.4 (0.1) | 0.615 |
| WBC, mean (SD) | | 5.5 (1.9) | 5.5 (1.9) | 5.4 (1.8) | 0.238 |
| WBC, n (%) | |  |  |  | 0.879 |
| Decrease | | 197 (11.4) | 160 (11.6) | 37 (10.7) |  |
| Normal | | 1454 (84.3) | 1160 (84.1) | 294 (85.2) |  |
| Increase | | 73 (4.2) | 59 (4.3) | 14 (4.1) |  |
| MCV, mean (SD) | | 93.1 (6.7) | 93.3 (6.6) | 92.3 (7.2) | 0.019 |
| MCH, mean (SD) | | 30.6 (2.7) | 30.7 (2.6) | 30.4 (2.8) | 0.125 |
| MCHC, mean (SD) | | 328.9 (12.2) | 328.8 (12.2) | 329.4 (12.2) | 0.394 |
| RDW-CV, mean (SD) | | 13.6 (1.4) | 13.6 (1.4) | 13.7 (1.4) | 0.114 |
| RDW-SD, mean (SD) | | 46.1 (4.5) | 46.1 (4.5) | 46.0 (4.7) | 0.570 |
| PLT, mean (SD) | | 145.2 (71.6) | 145.4 (71.1) | 144.6 (73.3) | 0.851 |
| PLT, n (%) | |  |  |  | 0.552 |
| Decrease | | 481 (27.9) | 379 (27.5) | 102 (29.6) |  |
| Normal | | 1174 (68.1) | 942 (68.3) | 232 (67.2) |  |
| Increase | | 69 (4.0) | 58 (4.2) | 11 (3.2) |  |
| NEUT%, mean (SD) | | 60.6 (9.9) | 60.5 (10.0) | 61.0 (9.4) | 0.374 |
| LYMPH%, mean (SD) | | 28.5 (8.7) | 28.6 (8.8) | 27.8 (8.3) | 0.131 |
| NLR, mean (SD) | | 2.5 (1.6) | 2.5 (1.6) | 2.6 (1.4) | 0.716 |
| MONO%, mean (SD) | | 7.6 (2.0) | 7.6 (2.0) | 7.9 (2.1) | 0.045 |
| EO%, mean (SD) | | 2.7 (2.3) | 2.7 (2.3) | 2.6 (2.4) | 0.673 |
| BASO%, mean (SD) | | 0.5 (0.3) | 0.5 (0.3) | 0.5 (0.3) | 0.967 |
| PT, mean (SD) | | 12.3 (2.8) | 12.3 (3.1) | 12.3 (1.1) | 0.730 |
| APTT, mean (SD) | | 30.1 (4.5) | 30.1 (4.5) | 30.2 (4.4) | 0.690 |
| TT, mean (SD) | | 20.3 (1.7) | 20.3 (1.7) | 20.3 (1.7) | 0.581 |
| FIB, mean (SD) | | 2.7 (1.0) | 2.7 (1.0) | 2.7 (0.9) | 0.539 |
| TBIL, mean (SD) | | 16.0 (15.2) | 16.1 (16.2) | 15.6 (10.4) | 0.432 |
| DBIL, mean (SD) | | 6.7 (12.4) | 6.7 (13.2) | 6.5 (8.5) | 0.749 |
| IBIL, mean (SD) | | 9.3 (5.0) | 9.4 (5.1) | 9.1 (4.6) | 0.257 |
| ALT, mean (SD) | | 47.0 (46.3) | 47.1 (46.9) | 46.6 (43.7) | 0.856 |
| AST, mean (SD) | | 49.0 (43.3) | 49.1 (43.5) | 48.7 (42.8) | 0.880 |
| A/A, mean (SD) | | 1.2 (0.7) | 1.2 (0.6) | 1.2 (1.0) | 0.767 |
| TP, mean (SD) | | 70.9 (6.0) | 71.1 (6.0) | 70.4 (5.8) | 0.036 |
| ALB, mean (SD) | | 42.3 (4.4) | 42.4 (4.5) | 42.0 (4.1) | 0.164 |
| GLB, mean (SD) | | 28.6 (5.0) | 28.7 (4.9) | 28.3 (5.4) | 0.249 |
| A/G, mean (SD) | | 1.5 (0.3) | 1.5 (0.3) | 1.5 (0.3) | 0.534 |
| ALP, mean (SD) | | 105.0 (59.7) | 105.2 (60.6) | 104.5 (56.3) | 0.839 |
| GGT, mean (SD) | | 98.7 (125.2) | 98.3 (124.1) | 99.9 (129.7) | 0.835 |
| GLU, mean (SD) | | 5.4 (1.8) | 5.3 (1.7) | 5.4 (1.9) | 0.451 |
| UREA, mean (SD) | | 5.5 (1.6) | 5.5 (1.7) | 5.4 (1.5) | 0.589 |
| CREA, mean (SD) | | 70.9 (15.2) | 71.0 (15.3) | 70.8 (14.4) | 0.808 |
| eGFR, mean (SD) | | 99.3 (14.4) | 99.4 (14.6) | 99.1 (13.7) | 0.700 |
| Cys-C, mean (SD) | | 1.0 (0.2) | 1.0 (0.2) | 1.0 (0.2) | 0.817 |
| URIC, mean (SD) | | 329.1 (78.4) | 329.8 (78.8) | 326.2 (76.6) | 0.437 |
| TG, mean (SD) | | 1.1 (0.7) | 1.1 (0.7) | 1.1 (0.7) | 0.742 |
| CHOL, mean (SD) | | 4.2 (1.0) | 4.2 (1.0) | 4.1 (0.9) | 0.035 |
| HDL-C, mean (SD) | | 1.3 (0.4) | 1.3 (0.4) | 1.2 (0.4) | 0.018 |
| LDL-C, mean (SD) | | 2.4 (0.8) | 2.5 (0.8) | 2.4 (0.7) | 0.199 |
| CK, mean (SD) | | 94.7 (152.6) | 97.2 (169.2) | 85.0 (43.5) | 0.018 |
| LDH, mean (SD) | | 200.4 (98.9) | 199.7 (97.3) | 203.4 (105.4) | 0.551 |
| HBDH, mean (SD) | | 153.6 (73.0) | 153.1 (72.4) | 155.5 (75.5) | 0.607 |
| TBA, mean (SD) | | 14.4 (24.5) | 14.0 (24.3) | 15.9 (25.2) | 0.197 |
| HBV DNA, n (%) | |  |  |  | 0.697 |
| Low | | 708 (41.1) | 570 (41.3) | 138 (40.0) |  |
| High | | 1016 (58.9) | 809 (58.7) | 207 (60.0) |  |
| HBV DNA Log, mean (SD) | | 3.0 (2.0) | 3.0 (2.1) | 3.0 (2.0) | 0.980 |
| CA19-9, mean (SD) | | 28.9 (59.6) | 29.4 (63.3) | 26.9 (39.7) | 0.353 |
| CEA, mean (SD) | | 2.8 (5.0) | 2.8 (5.4) | 2.7 (3.8) | 0.739 |
| CA-125, mean (SD) | | 23.7 (106.4) | 24.5 (118.5) | 20.4 (21.2) | 0.224 |
| AFP, mean (SD) | | 2178.5 (19502.7) | 2109.5 (18361.6) | 2454.0 (23546.5) | 0.800 |
| PIVKA-II, mean (SD) | | 8630.9 (19418.9) | 8914.3 (19945.0) | 7497.8 (17135.3) | 0.185 |
| AFP, n (%) | |  |  |  | 0.958 |
| <400 | | 1074 (62.3) | 859 (62.3) | 215 (62.3) |  |
| >400 | | 650 (37.7) | 520 (37.7) | 130 (37.7) |  |
| PIVKA-II, n (%) | |  |  |  |  |
| <2000 | | 827 (48.0) | 661 (47.9) | 166 (48.1) | 1.000 |
| >2000 | | 897 (52.0) | 718 (52.1) | 179 (51.9) |  |
| CA-125, n (%) | |  |  |  | 0.494 |
| <35 | | 1311 (76.0) | 1054 (76.4) | 257 (74.5) |  |
| >35 | | 413 (24.0) | 325 (23.6) | 88 (25.5) |  |
| HBsAg, n (%) | |  |  |  | 0.753 |
| Negative | | 282 (16.4) | 228 (16.5) | 54 (15.7) |  |
| Positive | | 1782 (82.9) | 1306 (83.0) | 476 (82.8) |  |
| HBsAb, n (%) | |  |  |  | 0.337 |
| Negative | | 1446 (83.9) | 1163 (84.3) | 283 (82.0) |  |
| Positive | | 278 (16.1) | 216 (15.7) | 62 (18.0) |  |
| HBeAb, n (%) | |  |  |  | 0.610 |
| Negative | | 390 (22.6) | 316 (22.9) | 74 (21.4) |  |
| Positive | | 1334 (77.4) | 1063 (77.1) | 271 (78.6) |  |
| HBeAg, n (%) | |  |  |  | 0.070 |
| Negative | | 1028 (59.6) | 807 (58.5) | 221 (64.1) |  |
| Positive | | 696 (40.4) | 572 (41.5) | 124 (35.9) |  |
| HBcAb, n (%) | |  |  |  | 0.902 |
| Negative | | 39 (2.3) | 31 (2.2) | 8 (2.3) |  |
| Positive | | 1685 (97.7) | 1348 (97.8) | 337 (97.7) |  |

## Supplementary Figures


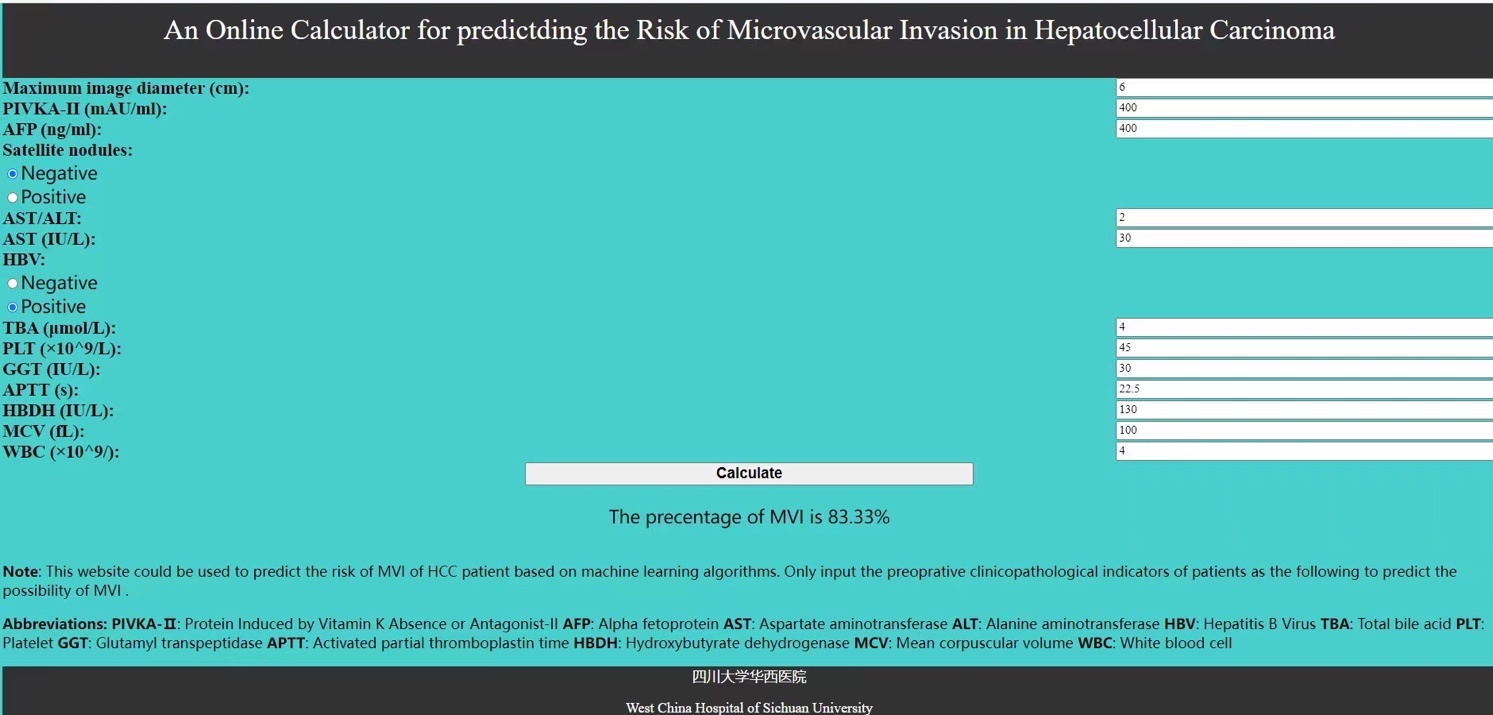


**Supplemental Figure** The online calculator for predicting MVI of HCC patients.
